# Attachment and grief in young adults after the loss of a close friend: a qualitative study

**DOI:** 10.1186/s40359-022-00717-8

**Published:** 2022-01-15

**Authors:** Iren Johnsen, Ane Martine Tømmeraas

**Affiliations:** 1 Center for Crisis Psychology, Department of Psychology, University of Bergen, 5007 Bergen, Norway; 2 Department of Psychology, University of Bergen, 5007 Bergen, Norway

**Keywords:** Adolescence, Young adult, Grief, Loss, Friend

## Abstract

**Background:**

Although many lose a close friend each year, they are seldom the focus of grief research. However, these losses often cause severe and long-lasting reactions. Deaths among adolescents and young adults are also often caused by traumatic events, e.g. from accidents, suicides, and homicides, placing them at significant risk for complicated grief reactions. The focus of this paper is bereaved friends after the shootings at Utøya, Norway in 2011, which is among a few studies that focus on bereaved friends, exploring how attachment affects the grief process after the loss of a close friend.

**Methods:**

This paper explores qualitative data from in-depth interviews with thirteen bereaved friends, conducted about 28 months after the loss. The interview sample consisted of eight females and five males, aged 18–31 years. The interviews were semi-structured, with a theme guide of 14 questions, and the method used for analyses was systematic text condensation.

**Results:**

Two main themes were identified from the analyses of the interviews: Friendship and Grief, with the subordinate themes: The importance of the friendship, Longing and remembrance, How the loss has affected other relationships, How the loss has affected the friend’s daily lives, Processing of the grief and Not being family. For most of the bereaved friends the loss and the grief had a profound effect on them and their overall lives, from daily functioning in school or at work, to changes in attitudes, and the way they were met as bereaved.

**Conclusions:**

The support, intimacy, and feelings of togetherness we share with our friends are of great importance and value for all people, but maybe especially for young people. When adolescents and young adults experience losses, their reactions are often intense and long-lasting, and especially complicated grief reactions can affect school performance and concentration, health, result in emotional problems; and disrupt development (e.g. identity formation and social skills). We don’t know much about the grief of bereaved friends and how their reactions can be explained. Thus, we hope that these findings could shed light on their grief reactions, and provide new knowledge on the short- and long-term psychological impact of losses of friends.

## Background

Losses among adolescents and young adults are relatively common (mostly grandparents), and 5% of Western adolescents may experience the loss of a close friend before the age of 15 years [1, 2]. Deaths among adolescents and young adults are often caused by accidents, suicides, and homicides, placing them at significant risk for traumatic bereavement occasioned by the traumatic deaths of their friends, romantic partners, and family members [3]. Sudden and violent deaths increase the risk of more intense and complicated grief reactions than do more natural losses [4, 5].

After traumatic losses it is common to experience preoccupation with the death, and also feelings of disbelief, anger, shock, guilt, depression, loneliness, sleep difficulties, emptiness, hopelessness, or vulnerability [6–9]. Prolonged grief has now been included in both DSM‐V [10] and ICD-11 [11] as a distinct mental disorder, and symptoms of prolonged grief include intense yearning and longing, preoccupation with the deceased, preoccupation with circumstances of the death, avoidance of reminders of the deceased or the death, trouble accepting the death, bitterness and anger related to the loss, and feeling meaningless [12]. Risk of complicated grief reactions and prolonged grief can be linked to younger age, and the type of relationship with the deceased, i.e., whether or not the bereaved was related to the deceased, and the specific nature of this relationship [13, 14]. The level of closeness in a relationship can be identified in features such as trust, intimacy, and mutual support [15].

Layne et al. [3] points to the interplay between posttraumatic stress symptoms and grief reactions that can arise following traumatic bereavement in adolescence. Traumatic grief, which often leads to complicated grief, is characterized by a combination of separation distress and traumatic distress, and there is often an association between complicated grief, posttraumatic stress disorder and depression [16, 17]. Exposure to the event even to a little degree may affect PTSD and complicated grief in young bereaved peers [16], and even those who don’t witness traumatic death circumstances themselves may generate distressing images and thoughts based on accounts from witnesses, family or images from the news [18, 19].

Adolescence and young adults who experience sudden and traumatic losses may often experience reactions such as shock, anger, guilt, concentration problems, sleep problems and vulnerability, in addition to preoccupation with the incident or the death circumstances [6–9]. Grief in adolescents and young adults can affect school performance and concentration, result in health problems, increased substance use, emotional problems, affect development and learning of social tasks [20, 21]. When it comes to gender differences, males may struggle to reconcile feelings of vulnerability and manly ideals of strength and stoicism, when they experience reactions like emptiness, anger, stoicism and sentimentality after the loss of a friend [22]. The grief experience and reactions of adolescence and young adults are affected by many things, e.g. the progression of developmental skills such as identity formation, coping availability and self-regulation [23], and they may also have new experiences and challenges of grief as they mature [24]. But also other factors have an impact, e.g. personality traits such as resilience and emotional reactivity, history of previous stressors and losses, available support systems, the circumstances of the loss and the relationship with the deceased [23, 25]. The period of adolescence and young adulthood is an important period for identity formation, e.g. exploring different identity roles, and the development of an autonomous self [24]. Young adulthood can be defined as the transitional stage of late adolescence or emerging adulthood [26, 27]. Adolescence and young adulthood include increased responsibility and maturity, and often involve a wish for more independence, separation, autonomy and freedom, e.g. from family and parents [28]. For adolescents and young adults, grief, and especially complicated grief, can disrupt development, e.g. identity formation and development of social skills [24, 29].

Herberman Mash et al. [30] investigated complicated grief in bereaved siblings and close friends and found that 16% met the criteria. Giannopoulou et al. [16] found in their study of traumatically bereaved peers after a bus accident that 21% had high levels of grief symptoms 18 months after the loss. Reactions after the loss of friends has long been an understudied area compared to reactions after other significant losses, although adolescents and young adults often experience intense and long-lasting reactions after these losses. These bereaved have, however, gained increased attention over the last years (e.g. [8, 30, 31], which may be a reflection of the increased recognition of friends as bereaved.

### Aims and research question

All though it is quite common to lose a close friend, this group of bereaved are seldom the focus of research on grief reactions. This paper explores qualitative data from interviews with bereaved friends after the shootings at Utøya, Norway, July 22^nd^, 2011, and is among a few studies where bereaved friends are identified as a unique group of bereaved.

The research question is: *How does attachment affect the grief process after the loss of a close friend?*

## Methods

In-depth interviews were used to gain an understanding of the lived experiences of the subjects.

### Aims, design and settings of the study

This study is based on data material from a larger longitudinal project “Bereaved parents, siblings and friends after Utøya July 22nd, 2011,” which aims to increase awareness of the bereaved’s situation after the killings at Utøya July 22nd, 2011. The study collected both quantitative and qualitative data, although only the qualitative data are used in this paper. Also, this paper focuses on the interviews with the bereaved friends.

### Data collection and sample

During the initial recruitment period from this project, parents and siblings of the deceased were asked to recruit four to six of the best friends of the deceased. In addition to participating in the survey part of the data collection, bereaved friends were asked to consent to participate in in-depth qualitative interviews. A theoretical sample was drawn from the total sample of bereaved friends participating at Time 1, and variation and breadth according to age, gender and geography was chosen as the criterion for sampling. Based on previous research on populations bereaved by potentially traumatizing deaths, a careful and respectful approach was emphasized [32] and every stage of the research was carried out in a very sensitive and careful way, showing deep respect for the possible pain experienced by the bereaved. The project was approved by the Regional Committees for Medical and Health Research Ethics (REK) in Norway. Data collection was performed in accordance with the Helsinki Declaration, and all interviews were anonymized.

The interview sample consisted of 13 young adults: eight females and five males, aged 18–31 years (mean = 21.77, *SD* = 3.70). 54% of the participants had lost a male friend (*n* = 7), while 39% (*n* = 5) had lost a female friend and one had lost a girlfriend. One of the informants had lost two friends.

### Data collection and analysis

Thirteen interviews were conducted about 28 months after the loss. The interviews took place in the informants’ house, or at a place of their choice. The interviews were semi structured with a theme guide of 14 questions to ensure that certain subjects were addressed systematically: (a) their experiences of the loss, and how it had affected their life, (b) circumstances concerning the death and how this had affected their grief, (c) help and support after the loss, (d) functioning at school or work and (e) self-coping. This article’s focus, however, is only on how the friendship or attachment impacted their grief, so certain parts of the interviews were deemed more relevant. During the interviews it was important to allow the friends to generate their own stories, and the theme guides were used as a starting point with follow-up questions depending on how the individual interviews developed. Each interview lasted approximately 2.5 h (ranging from 1.5 to 3.0 h).

All interviews were audiotaped, transcribed verbatim, and analysed using systematic text condensation [33]. Because the focus of this article is narrower than the scope of the interviews, certain parts of the interviews were used for further analysis, while parts that were not relevant did not get used. Systematic text condensation consists of (1) reading the data material to get an overview of important themes, (2) identifying and sorting meaning units into code groups, (3) sorting the meaning units into subgroups and reducing these into condensates and (4) using the condensates to develop descriptions and concepts. To handle the large amount of data and to sort the meaning units, NVivo was used.

## Findings

To answer the research question two main themes were identified from the analyses of the interviews: Friendship and Grief. To deepen our understanding of the friendship and attachment, three subordinate themes will be described: the importance of the friendship, longing and remembrance and how the loss has affected other relationships. Regarding the main theme of grief, these three subordinate themes will be described: how the loss has affected the friend’s daily lives, processing of the grief and not being family.

### The importance of the relationship

The friendship with the deceased and its importance impacted everything the participants of the study talked about. This relationship was and is still important to the young adults, and the loss of the friend has had a profound impact on them. The value of this friendship was expressed through the way the bereaved friends talked about the deceased’s character, and the way they described their friends with admiration and affection. They talk about their friend as someone “unique”, “positive” and “warm”, as for this young woman: “There's not a lot of people like her, so thoroughly good. And I feel like I try to be like her or… that it makes me a better person to have that with me.” They also talk about characteristics that are hard to find in other people or other relationships, like their kindness and good values. This is a person it is sad to not have in their lives anymore.

Many of the bereaved friends also expressed the value of the friendship through describing what they used to do together. Some talked about always spending time together, while others described constantly texting or how it was always very special every time they met even though they did not live in the same city or spent time apart. Many of the friends also talked about having a similar sense of humour, laughing together, and having a good time, and the small things that make a friendship—common hobbies, cooking together, the communication. A big part of this was the importance of having someone to “talk to about anything”.

The significance of the friendship becomes clear in the way they describe what was so special about this friend, and this particular friendship. The bereaved friends often describe the deceased as their “oldest friend” or “the closest friend”, and a person that was important in their lives: “I thought of him as my closest friend (…), it was him I shared everything with”, like this young woman said. This was the type of relationship “you cannot explain with words”, and a person to be “fully comfortable around”—someone who is always there for you. Frequently used descriptions were “closeness”, “feeling of understanding” in the friendship, someone to “turn to” in difficult times. The friendship is also compared with the other friendships they have (both good and bad), like this young woman: “I have many friends, but nothing will be the same as that friendship”. They express gratitude for having had this type of close friendship, like another young woman said: “I am very, very thankful for the time I got to know her, but at the same time it is horrible that I did not get to know her for a longer time.”

Many struggled with knowing that in the future, their friend would not be there, and that no one would take the friend's place in their life. This important role in their lives will never be filled again, because no one could ever replace their friend—something several of the friends thought about: “He is not replaced, and he never will be”, said one young man. Some of the friends also expressed sadness because people they meet in the future will not get to know the deceased friend, that people will not know how good this person was and their importance, like one young woman says: “It is sad to think about the people I will meet in the future who will not know who she was”.

### Longing/remembrance

The bereaved friends describe longing and remembrance of their friends, something they experienced partly through feelings of loneliness and emptiness. After the passing of their friend many of the young adults described situations and occasions where it became especially apparent that their friend was gone, e.g., not being able to call or text if they had something important to say. Like this young woman says: “Who am I going to talk to? Who is going to send me a happy message saying that they are in love? I feel like so much is missing, without being able to explain it.” Some described situations where they found themselves trying to call or dial their number, only to remember that they cannot do that anymore, as this young man described: “I know that no one would answer, and she always used to answer (…) I miss it all the time, meeting her when I came home, because I remember how nice it used to be.” When they could not spend time with or talk to the person they preferred doing so with, many said they perceived life as as “empty” and “meaningless”.

The loss also became evident in the things that reminded them of their friend and that they were gone. Several experienced at times forgetting that their friend was gone, because they were so used to having that person in their lives. However, many things did make them remember the loss, as described by this young woman: “I am reminded of it when her brother comes by. And I am reminded of it when, I mean, there is always an empty seat when we all get together, there is always one piece of cake left”. Many of the bereaved friends have had experiences where they saw someone and thought it was their friend—something that also made them aware of the reality and the loss.

### How the loss has affected other relationships

Several of the bereaved spoke about struggling with social relationships after the loss and not enjoying social situations like they used to. This caused some of the bereaved friends to withdraw from social life. As for this young woman: “When I am hanging out with friends, and it is the middle of summer and everyone is happy, and I just sit there and feel like I am not able to participate in the discussion or talk, because I do not want to, I am struggling because I miss them and only want to be with them.”

A few of the bereaved friends expressed not wanting to share their grief with the people around them, and in effect keeping it to themselves. This could be due to feelings of “bothering others” with their emotions or feeling like they should be able to “handle it themselves”. One young woman said this: “I feel like there is no space for it, no space in the friend group, at work, almost no space in the family (…) You have to take that space and say that you need to cry because you are sad. But you do not take that space, because you fear the reactions.” Others more actively or passively sought out or found comfort in other people and seeing that friends cared and became closer “felt good”. One man talked about realising that having other good friends helped and could fill some of the emptiness after the loss.

### How the loss has affected the daily lives

Many of the bereaved friends talked about how the loss and subsequent grief impacted their daily functioning—it affected them in their work, schoolwork, and overall lives. They explained that suddenly going back to reality was difficult; Some isolated themselves or struggled with not being able to do anything. Others went back to school or work but were not able to perform the way they wanted or expected to. Functioning became difficult due to reminders or constant rumination, “noise that never stopped”, and grieving the loss also made it hard to be present in a class or work setting, due to feelings of sadness. Problems with sleep also made it hard to concentrate at school or work, like this young woman said: “I struggled with sleeping and woke up several times a night and could not wake up once I did fall asleep. It was tiring when I had to go to school and knew I had to get up a few hours later, so I could not sleep at all.” Some described more serious issues, like dropping out of school. For some these impairments of functioning led to anger, like this young woman says: “Things got so hard I could not finish school. This is something I have been very angry about, because it was my last year, I was angry that I could not make it work, and that they could not help me better.”

Related to the impairments of daily functioning and the mental exhaustion, some of the bereaved friends also experienced physiological or physical reactions after the loss, such as panic reactions, hyperventilating and feeling like they couldn’t breathe, as well as constant stress. One young woman struggled with self-harm and issues surrounding food after the loss. Some describe sleepless nights thinking of or dreaming about their deceased friend, others experienced nightmares, while some could sleep normally. One young man remembers struggling with dreams after the loss often involving weapons, and one young woman said she wished that she had also died that day. Others described a constant fear of death, for themselves or the people they love.

### Processing of the grief

The bereaved friends have had to learn to live with the grief. For some it was hard processing the loss because of the event surrounding the death itself; in a way this grief “belonged to the country”, like one young woman says: “I think it would be easier to accept if it was another setting.” Several of the friends expressed concern with not being able to move on and being stuck in the grief process, like this girl: “I can’t fully accept that I am so far behind where I thought I would be. I don’t know if I misunderstood, but I thought I would have moved on more, I thought it would be easier, and I get frustrated when it’s not.” She further talked about how it is going to take a “long time before it gets easier.”

For many the loss has gotten better with time, and the young adults describe simultaneously being happy and unhappy, learning to live with the grief, and knowing that it will always be there, like a young woman says: “I have made a rule for myself, to not only associate July 22nd with grief, but love and laughter, because we shared so many nice moments”. They still remember the good times with their friend and express gratitude for having known this person, and even though they still miss them, the grief does not feel as heavy anymore. One young man says: “The grief is not as present anymore, but it is gradually being replaced, sometimes there are good feelings, I think it is gradually turning into memories.” Many of the friends talked about their attempts to try to move on with their lives, as one young woman says: “I can cry a little, and then I have to swallow it and continue with the day”; Another one describes it like this: “You remember how hard things were, and you have seen it in the people around you how hard it was, but you have to function, you have to wake up, you have to breathe, if not, you would die”.

In processing the grief, some of the young adults talked about the loss making everything seem meaningless, while others gained insight about themselves and their life. One young man says: “You want a loss to mean something (…), it has to mean something, if not it is meaningless, I mean, I think it is meaningless, I do.” Several of the bereaved friends felt that there was little joy left in the world without their friend, and that it would make no difference if they also died. Some of the friends experienced feelings of anger, not being able to reason or understand, like this young woman describes: “How are we going to love lots of people for them to just die, it's grotesque”. This can also be related to the nature of the death, like one young woman says: “She was so young, and had so much, if she had died in a traffic accident, I think that would be easier to understand, rather than that a person shot her in the head.” The bereaved friends struggled with understanding not only that they had lost their closest friend, but also that they had been killed. This led to despair and hurt. Several of the bereaved friends described feeling worthless when the world eventually moved on.

For others the loss and grief made them realise what was important to them and described the grief turning into healthy longing to help them be conscious and grow as a person, such as appreciating the smaller things in life. As one young woman says: “I have never been religious or anything, but I tried to find my own belief, find out what happens when you are gone, and tried to find solace in everything. I tried to look at the bigger picture, we are so small on this planet, and when we are gone, it is like it was before birth, or we just disappear, out into eternity. Maybe it is absurd, but I found comfort in that.” This perspective led some to realise that life is short, that they must live the best life they can, share the good and the bad times with other people, and take care of the people they love. They describe having realisations of never knowing if you get another day, of being made aware of their own mortality in a new way. But this awareness of mortality did not seem to scare them, and the insight was associated with something positive, something to make them appreciate life in a new way. One young woman says this made her think about choices in a new way, of not being afraid of consequences: “When I have had to make decisions the last few years, I have thought more about not focusing on the long-term. because I do not know how long I will live. I think about how suddenly it happened for those who were at Utøya, and that it could happen to me too, it is something I feel all the time, I do not know how long I will live.”

Many of the bereaved friends describe grieving as something unpleasant, like this young woman: “It is not something you want to feel (…) it is like jumping into a pool, you just, you don’t get yourself to do it, you know it's uncomfortable.” However, they could not avoid grieving, as described by this young woman: “It was very hard, but at the same time I was focused on having to get through it, it was not something I could swallow and bring up again a year later.” Many of the friends talked about actively focusing on getting through the grief by seeking comfort alone, in family, with friends or priests, or by talking about the loss, reading about the events, or going to the cemetery. For some, actively taking the time or place to grieve made it easier to go back to reality again without always getting distracted. Some of the bereaved friends explained that there was a comfort in taking the time to grieve and remember their friend, in knowing that they will not forget them, but “keeping them alive in their mind”.

### Not being family

Even though the participants of this study were very close to the deceased, lack of recognition of friends as bereaved had implications for how they were met by professional helpers and other people, as well as how they saw themselves in relation to the loss. In some ways the friends felt forgotten, and not entitled to grieve in the same way as family or an “inner circle” of those affected. They especially experienced this in public condolences or during the funeral, where the focus was mostly on the family. One young woman expressed doubts concerning the closeness of the friendship, like she was not allowed to grieve: “You felt like, maybe you were overreacting, because everybody, no one thought you had suffered a loss, so why did you? Was it really that bad?”.

They felt that other people did not see them as part of the affected”, including getting help from professionals, compared to what the family was offered. After the killings the families were offered a lot of help and assistance, but the friends experienced not being offered anything, as this young woman says: “I feel like the closest family has gotten a lot, a lot of help, they still get help, all the help they want. But everyone else, there are so many who are suffering, who has not gotten any help at all.” Many of those who lost a friend experienced being put aside, not getting the help they could have needed—and even though they rationalise it and express understanding for regarding resources to the families, some of the bereaved friends in the study thought it “could have helped to be recognised as suffering from the beginning.”, as described by a young woman. Another young woman says: “I have understood that I am affected, but I am not bereaved.”

In addition to being forgotten or not receiving help, it seems like some of the friends don’t recognise themselves as being bereaved, even though they clearly are suffering and grieving the loss of their friend. They talk about how they “by definition” do not consider themselves as being bereaved, and that word being exclusive to the family, like this young man: “You can love them like family, but it is something different, filling the void after a friend is easier than filling the void after a lost brother. It will never be the same, there is a difference.”

## Discussion

For most of the bereaved friends the loss and the grief had a profound effect on them and their overall lives, from daily functioning in school or at work, to changes in attitudes, and last the way they were met as bereaved. In this part we will discuss our findings in regard to theory and to current and previous research in the field. First, we will discuss how the loss affected the bereaved friends and the characteristics of their grief, and then we will explain this by the close relationships these young adults had with their friends.

### The loss affected their whole life

When adolescents and young adults experience death of friends it is often caused by violent and sudden deaths after accidents, suicides, and homicides [3], which increases the risk of more intense grief reactions compared to more natural and expected losses [4, 5]. The bereaved friends reported many problems after the loss, like strong feelings of sadness, reminders and rumination, which caused problems with, i.e. concentration, and sleeping problems and/or nightmares. Related to the mental exhaustion, some of the bereaved friends also experienced physical reactions, like panic attacks, hyperventilating and feelings of constant stress and activation. Many of the friends struggled with anger and rumination about the way the death happened, and that their friend had been shot, and this made processing the loss harder. This is also found in other research, and it is common to experience, e.g. preoccupation with the death and the circumstances of the death, disbelief, anger, bitterness, shock, trouble accepting the loss and guilt after traumatic losses [6–9, 12]. Thus, following traumatic losses, bereaved may experience both posttraumatic stress symptoms and grief reactions [3, 17]]. This confirms the symptoms that the bereaved friends in our sample experienced, i.e. activation and arousal. Giannopoulou et al. [16] found that exposure to the event even to a little degree affected PTSD and complicated grief in young bereaved peers. This confirms that even those who don’t witness traumatic death circumstances themselves may generate distressing images and thoughts, based on accounts from witnesses, family or images from the news [18, 19]. Rumination about the death and the deceased may lead to poorer adjustment to the loss, and a more complicated grief processing [34].

Several of the friends expressed feelings of emptiness, meaninglessness and lack of joy in their life, which is also common to experience after traumatic losses [6–9]. Many of the friends also felt they were stuck in the grief, and that it was hard to move on, especially when the rest of the society moved on. This may have been especially hard to handle, since in the beginning the whole society seemed to feel “ownership” of this event, and some felt that the grief “belonged to all of Norway”. However, the society moved on quite quickly, while they continued to grieve. Some also explained that it would have been easier if the death had happened under other circumstances. According to Servaty-Seib and Pistole [35], time since death is not a straightforward predictor of grief intensity for adolescents, and that the further adolescents are from the loss, the greater they perceive their past grief intensity to have been. This could be explained by: (a) adolescents idealize their retrospective accounts; (b) as they move beyond the initial grief, they realize how upset they were initially, and appraise and report the past grief more accurately; and (c) the memory of the past grief is stronger in comparison to present grief. Liu et al. [31] found that loss of a friend caused significant adverse physical and psychological well-being, poorer mental health and impairment in social functioning, which occur up to four years following bereavement. Having to go back to reality, both to work or school or socially, often without being able to perform or function the way they wanted and expected, was difficult for many of the bereaved friends in our sample. Due to this many felt anger, towards themselves or school, while others isolated themselves. Some of the bereaved friends struggled with social relationships after the loss and had withdrawn from social life. This was explained by not having fun in social settings anymore, not wanting to be parts of larger groups, and experiencing reminders of their friends. Social deprivation and social withdrawal can however prolong the deterioration in physical and mental health after a loss [31, 36]. The internal structure and distinctiveness of CG in adolescents may bear resemblance to what is known from adult populations, but focus on depressive and anxious symptoms in bereavement-related distress might overlook that other patterns of complications may occur in bereaved adolescents [6]. According to Kokou-Kpolou et al. [37] the death of an immediate family member can be associated with higher levels of PCBD-separation distress, while the traumatic death of a friend and romantic partner can be more associated with PCBD-social and identity disruption. Going through both positive and negative life events (e.g. developing personal relationships and learning new roles, but also e.g. breakups with friends and partners) are important for development through fostering confidence, independence and self-efficacy. Experiencing something traumatic in this period, that leads to impaired functioning either social or at school or work, may imply that important lessions are not being learned [38].

Although it was hard in the beginning, they learned to live with the grief, and they saw that things got gradually better. They knew that the loss would always be with them, but they learned to appreciate the time they got with their friend and express gratitude for the time that they had together and knowing that they would always remember them. Many of the bereaved friends also described gaining new insight about themselves and life, developing as a person and growing with the experience. Through processing the loss, they realized what was important to them, made them more conscious and appreciate life more. This made them try to live their life in the best way, and spend time with and care for the people they love. Actively taking time to grieve also made it easier to go back to reality.

To many bereaved peers both parents and peers are seen as being most helpful, with peers found to be quite helpful in all situations, while teachers and school counsellors are seldom seen as being helpful, according to Ringler and Hayden [9]. Many friends in our sample also sought comfort in family and friends. All though many of the bereaved friends received good help and support from their network, some expressed that they felt that they should be able to handle it on their own and didn’t want to bother others with their problems. This made them keep their emotions to themselves. The duration of help received is often below what bereaved adolescents and young adults want, although a longer duration of help seems to be tied to a more positive present adjustment [35]. Often bereaved peers just want someone to understand their feelings and listen to them [9]. Support from friends and peers can be lacking because of many reasons, e.g. inexperience or lack of knowledge, or discomfort and insecurity. This may however give the bereaved feelings of not being understood and isolated, which then can complicate the grief processing [39]. Despite the close relationship these young adults had with their friend, many of them were met with lack of recognition as bereaved, both by professionals and others. They expressed understanding for family coming first, but this made them feel forgotten, not entitled to grieve, and not getting the help that they needed and wanted. These attitudes also affected their own perception of themselves as bereaved and made some of them doubt their own feelings and reactions.

### Closeness in the relationships intensify the grief reactions

All though many adolescents and young adults lose their close friends every year, we don’t know as much about this bereaved group and how their reactions can be explained, as we know about other groups of bereaved. Other studies have found strong reactions in this group also, i.e. Herberman Mash et al. [30] who found the prevalence of complicated grief in bereaved friends to be 16%, and Giannopoulou et al. [16] who found in their study of traumatically bereaved peers that 21% had high levels of grief symptoms 18 months after the loss. The prevalence of complicated grief in the total sample of bereaved friends in our study was however 69% 3.5 years after the loss, which was the same time as the interviews were conducted [40].

The way the bereaved friends talked about the deceased and expressed the value of the friendship, showed the importance of the relationship, and the appreciation of their dead friend was clear in everything the young adults talked about. The quality of the relationship with the deceased and the level of closeness can be important for the intensity of the grief reactions [25, 35, 41]]. Closeness in a relationship includes features like trust, intimacy, and mutual support [15]. In the total sample of our study, the mean of the self-reported evaluation of the closeness of the relationship (ranging from 1 to 10) was 8.89 [42]. Complicated grief and somatic symptoms can be related to the quality of the relationship (depth/conflict) with the deceased friend, and those who reported greater depth in their relationship were more likely to have complicated grief [40], and emotional closeness to the deceased can cause more intense grief reactions [25, 43]]. This corresponds well with the findings of our study, where many of the bereaved friends talked about longing, and feelings of emptiness and loneliness, and that the loss became especially evident in different situations and occasions. They were so used to having this person in their lives, therefore so many small and bigger things made them remember the loss. They missed the things they used to do together, just hanging out, having a good time or constantly texting, basically the small things that make a friendship. Most of all they missed someone to “talk to about anything” or turn to in times of trouble. The level of positive relationship quality and satisfaction has been found to be related to increased feelings of yearning and experiences of loneliness [44]. Servaty-Seib and Pistole [35] also found that present grief was significantly higher for loss of close friend than for grandparent loss, and that both prediction of past and present grief was affected by emotional closeness to the deceased.

During adolescence and young adulthood many lasting relationships are established, and one can see a strong influence of peers in many areas of life [27]. The bereaved friends in our sample were sad that they would not have a future with this person, and that people they would meet in the future would not meet their friend and get to know how great they were. Some also talked about feeling that this important friendship role never would be filled again, because none of their other friends could take the friend’s place in their life. During adolescence, and into young adulthood, is an important period of identity formation, with increased responsibility, maturity, independence, separation, autonomy and freedom, e.g. from family and parents [24, 28]. When adolescents and young adults experience loss in this period, they are often deeply affected, and their lives may change forever [9].

## Conclusion

The support, intimacy, and feelings of togetherness we share with our friends are of great importance and value for all people, but maybe especially for adolescents and young adults. This period of life is important for many developmental issues, e.g. identity formation, maturity, independence, separation from family and parents, and autonomy. When adolescents and young adults experience loss in this period, they are often deeply affected, and their lives may change forever.

It was clear that for most of the bereaved friends the loss and the grief had a profound effect on them and their overall lives, from daily functioning in school or at work, to changes in attitudes, and last the way they were met as bereaved. The way the bereaved friends talked about the deceased and expressed the value of the friendship, also showed the importance of the relationship, and the appreciation of their dead friend was clear in everything the young adults talked about. The quality of the relationship with the deceased and the level of closeness in these relationships, especially for the bereaved girls, could be a possible explanation for these findings. All though many adolescents and young adults lose their close friends every year, we don’t know as much about this bereaved group and how their reactions can be explained, as we know about other groups of bereaved, and we hope that these findings could shed light on the grief reactions and symptoms of this group of bereaved.

### Implications of the findings

All though there has been some increased attention on the loss of close friends over the years, this bereaved group is an understudied area compared to other groups of bereaved. This is among a few studies that has identified bereaved friends as a unique group of bereaved, and that focus solely on their reactions. This may lead to more knowledge and recognition on what characterizes this type of grief compared to grief after other losses. Adolescents and young adults often experience intense and long-lasting reactions after these losses, and especially complicated grief, can disrupt development, e.g. identity formation and development of social skills [24, 29]. This can have consequences like impaired school performance, health problems, emotional problems, and can also affect development and learning of social tasks [20, 21]. The study of bereaved friends’ reactions and grief provides new knowledge on the short- and long-term psychological impact on this group.

Bereaved friends can have severe and long-lasting reactions after a loss, and should be recognized as a group of bereaved, so that they can receive the help and support that they need [31]. Servaty-Seib and Pistole [35] highlight the importance of researchers and professionals intentionally and directly assessing bereaved adolescents' perceived emotional closeness to the deceased as part of grief-related counselling, and to consider attachment, when examining closeness in adolescent friend and kin relationships.

## Data Availability

The datasets generated and/or analysed during the current study are not publicly available, but are available from the corresponding author on reasonable request.
